# Visceral Leishmaniasis and HIV Coinfection in East Africa

**DOI:** 10.1371/journal.pntd.0002869

**Published:** 2014-06-26

**Authors:** Ermias Diro, Lutgarde Lynen, Koert Ritmeijer, Marleen Boelaert, Asrat Hailu, Johan van Griensven

**Affiliations:** 1 Department of Clinical Sciences, Institute of Tropical Medicine, Antwerp, Belgium; 2 Department of Internal Medicine, University of Gondar, Gondar, Ethiopia; 3 Public Health Department, Médecins Sans Frontières, Amsterdam, the Netherlands; 4 Department of Public Health, Institute of Tropical Medicine, Antwerp, Belgium; 5 School of Medicine, Addis Ababa University, Addis Ababa, Ethiopia; National Institute of Allergy and Infectious Diseases, United States of America

## Abstract

Visceral Leishmaniasis (VL) is an important protozoan opportunistic disease in HIV patients in endemic areas. East Africa is second to the Indian subcontinent in the global VL caseload and first in VL-HIV coinfection rate. Because of the alteration in the disease course, the diagnostic challenges, and the poor treatment responses, VL with HIV coinfection has become a very serious challenge in East Africa today. Field experience with the use of liposomal amphotericin B in combination with miltefosine, followed by secondary prophylaxis and antiretroviral drugs, looks promising. However, this needs to be confirmed through clinical trials. Better diagnostic and follow-up methods for relapse and prediction of relapse should also be looked for. Basic research to understand the immunological interaction of the two infections may ultimately help to improve the management of the coinfection.

## Introduction

**Figure 1:**
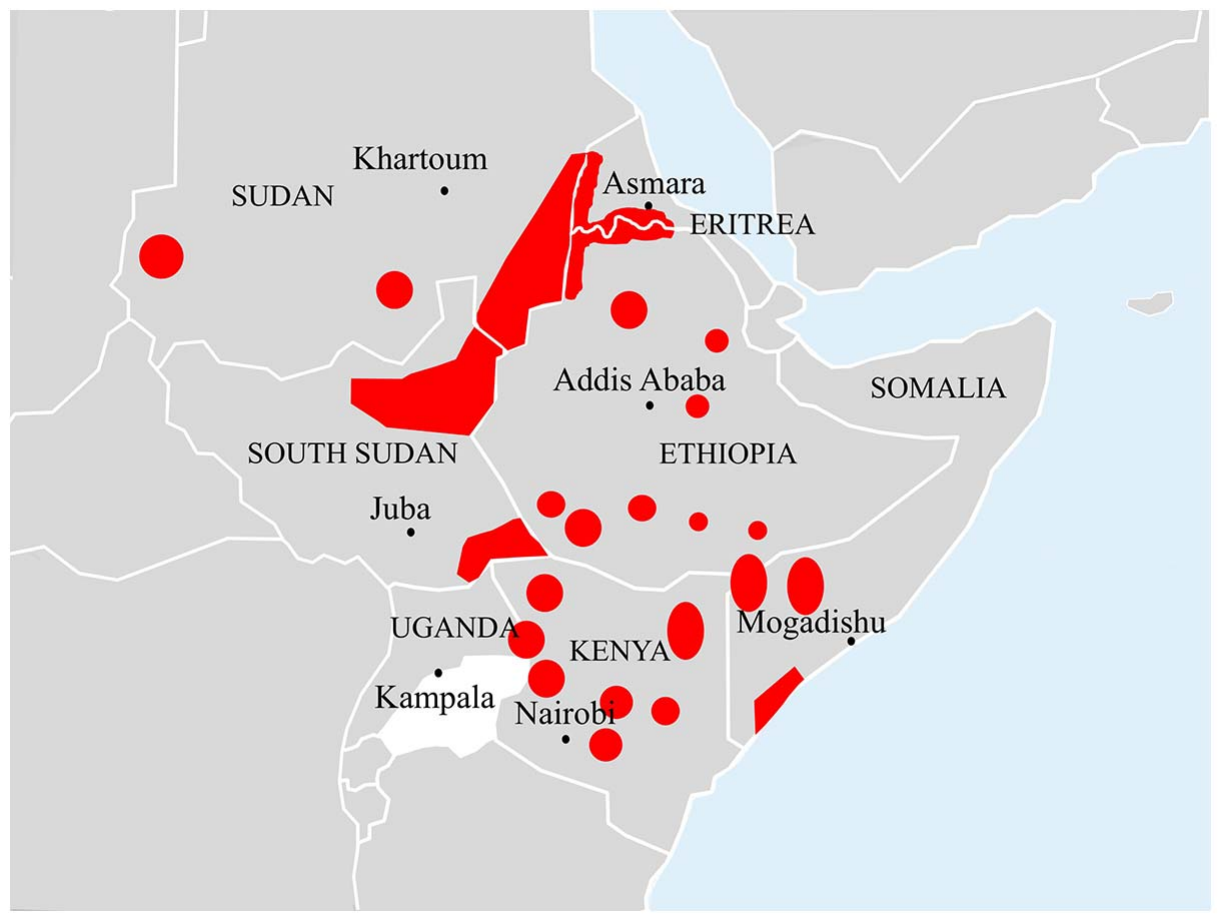
Map of East Africa showing the geographic distribution of visceral leishmaniasis. Map taken from “Malaria Consortium; Leishmaniasis control in eastern Africa: Past and present efforts and future needs. Situation and gap analysis, November 2010” [15].

Visceral leishmaniasis (VL) is a vector-borne protozoan infection targeting the reticuloendothelial system [1]. Its occurrence is widespread, being prevalent in approximately 70 countries worldwide. East Africa is one of the most affected regions, second only to the Indian subcontinent, with an estimated annual incidence rate of 29,400 to 56,700 cases [2]. The countries most affected in this region are Sudan, South Sudan, and then Ethiopia. Although with much lower VL burden, endemic foci of the disease are also found in Eritrea, Somalia, Kenya, and Uganda [2]. Figure 1 shows the VL-endemic regions in East Africa. The disease typically affects poor communities residing in remote places with poorly functioning health-care systems.

Historically, East African VL has claimed the lives of many people, with the most infamous epidemic reported from South Sudan by Seaman et al. [3]. Between 1984 and 1994, a devastating epidemic in the western Upper Nile region in South Sudan claimed the lives of an estimated 100,000 people [3]. To date, treatment and care for VL in these resource-poor countries is mainly provided or supported by international organizations such as Médecins Sans Frontières (MSF), Drugs for Neglected Diseases initiative (DNDi), and the World Health Organization (WHO).

The simultaneous infection of humans by HIV and *Leishmania* almost always leads to a “deadly gridlock,” as they both have the same deleterious effect on the immune response [4]. The majority of VL-HIV coinfections were previously reported from the Mediterranean countries during the pandemic years of HIV/AIDS in the 1990s, with the prevalence of HIV among VL patients reaching up to 60% in intravenous drug users in Spain [5], [6]. As a consequence of HIV coinfection, atypical presentations of VL, a high rate of treatment failure, and frequent relapses were reported. Only after the introduction of antiretroviral therapy (ART) was a decline in incidence of VL-HIV coinfection observed [5]. With the spread of HIV to other VL-endemic regions of the world, the coinfection is now reported from 35 countries [5], [7]. Because of the alteration of the disease course, the diagnostic challenges, and the poor treatment response, VL with HIV coinfection has become a very serious challenge in East Africa today.

### HIV Epidemiology in East Africa

The prevalence of HIV increased alarmingly from the mid-1980s to the 1990s in most African countries. Since 2000, however, a decline has been seen in the number of new HIV infections, which can be explained by a variety of factors, most notably preventive measures and access to ART [8], [9]. In Ethiopia, HIV prevalence has declined from 5.6% in 2005 to 2.6% in 2011 (antenatal care sentinel surveillance [10]), and the estimated prevalence among the adult population is 1.5% (Demographic and Health Survey [DHS] 2011) [11]. However, despite the decreasing prevalence of HIV in the general population, the prevalence of HIV among VL patients has remained proportionally very high. The northwest districts of Ethiopia along the Sudanese border report the highest burden of HIV and VL coinfection rates, with HIV prevalence rates of 20%–40% among VL patients [5], [7], [12]. The 2012 annual report from the Leishmaniasis Research and Treatment Centre of the University of Gondar showed that 81/332 (24.4%) of all admitted VL cases were HIV coinfected (unpublished data). The rates of coinfection from different studies in Ethiopia are summarized in Table 1.

**Table 1:** Reports of HIV coinfection rates among VL patients in East Africa.

Country	Study Reference	Place, Study Period	Sample Size	HIV Coinfection Prevalence	Overall VL Case Mortality	HIV/VL Case Mortality
Ethiopia	Hailu A [64], 2006*	Addis Ababa, Army Hospital, 1992–2001	291 soldiers	48.5%		
	Ritmeijer K [23], 2001γ	Humera, NW Ethiopia 1998–1999	145 migrant workers	18.6%	24.1%	33.3%
	Lyons S [65], 2003γ	Humera, NW Ethiopia, 1998–2000	213 migrant workers	23.0%	11.3%	26.5%
	Ritmeijer K [31], 2006γ	Humera, NW Ethiopia, 2004	375 migrant workers	28.5%	Miltefosine: 2.1%, SSG: 9.7%	Miltefosine: 1.6%, SSG: 6.8–19.3%
	Mengistu G [12], 2007*	Gondar hospital, NW Ethiopia, 1999–2004	212 migrant workers	41%	24.4%	39.3%
	ter Horst R [28], 2009γ	Humera, 2006–2007	128	34.4%		
	Hurissa Z [19], 2010γ	Gondar+Humera 2006–2008	241	38.2%	10%	17.4%
	MSF, unpublished dataγ	Abdurafi, NW Ethiopia	migrant workers			
		- 2008	186	20%		
		- 2012	298	11.1%		
South Sudan	MSF, unpublished data	Greater Upper Nile, 2001	488	0.4%		
	MSF, unpublished data	Greater Upper Nile, 2010–2012	2,426 (62% <15 years)	2.5%		
Sudan	MSF, unpublished data	Gedaref, 2010–2013	1455 (71% <15 years)	1.3%		
Kenya	MSF, unpublished data	Western Pokot, 2006–2012	1595 (63% <15 years)	1.4%		

Abbreviations: MSF, Médecins Sans Frontières; NW, northwest.

γ : reports on primary VL;

*: reports in all VL cases.

The particularly high HIV coinfection rate in northwest Ethiopia could be due to the massive population movement in the region [13]. In this area of cash-crop farming, there is a high labour demand, and 300,000 to 500,000 highlanders from urban and semiurban areas seasonally move in and out of the region. When these *Leishmania*-nonimmune highlanders go to the VL-endemic regions, they become exposed and infected. Internal migration is also a risk factor for HIV, and those infected with HIV will develop overt disease (VL) more rapidly than those who are not infected [5]. The HIV prevalence rate among migrant workers at the MSF Holland voluntary counselling and testing services at Humera and Abdurafi was 8%–12% during 2008 and 2012 (MSF, unpublished data). Over the period from 2009 to 2012, MSF treated 1,255 primary VL cases, out of which 235 (18.7%) were HIV positive. The HIV prevalence among primary VL cases in the other MSF treatment areas in the same period were as follows: South Sudan 61/2,426 (2.5%); Sudan 19/1,455 (1.3%); and Kenya 22/1,595 (1.4%).

In Sudan, a hospital-based study in Khartoum reported a coinfection rate of 9.4% in 2002. Reports from southeast Sudan (Gedarif state), a region characterised by population movements, found HIV coinfection in 3.6% of VL cases in 2003 [5]. Systematic testing of VL patients for HIV in MSF programs in Sudan and South Sudan between 2009 and 2012 showed an HIV coinfection rate of 2%–2.5% (MSF, unpublished data). In the other neighbouring countries and endemic regions, mainly children are affected, and the HIV coinfection rate is very low [5]. MSF experience in Kacheliba in Western Pokot, Kenya, showed 1.4% HIV coinfection rate between 2006 and 2012. However, only 1,595 of the total 3,007 primary VL cases during the period were tested for HIV (MSF, unpublished data).

### VL Etiology

VL in East Africa is caused by *Leishmania donovani* and is transmitted by a sand fly of the species *Phlebotomus orientalis* or *P. martini* in a predominantly anthroponotic cycle (i.e., from man to man without animal reservoir) [14], [15]. Although less virulent parasites (nonhuman pathogenic trypanosomatids and etiologic agents of the cutaneous form of the disease) were isolated from VL cases in some HIV-coinfected cases in zoonotic transmission regions [5], there are no reports of such cases from this region to date.

### VL Clinical Manifestations

Patients with VL typically present with prolonged fever, weight loss, prostration, splenomegaly, and pancytopenia [1]. While these manifestations are common for both non-HIV and HIV coinfected patients, patients can present with atypical clinical symptoms in cases of severe immunosuppression. *Leishmania* parasites, which are normally concentrated in the reticuloendothelial organs, are often disseminated, and high parasite loads can be found in peripheral locations such as the skin, gut, lungs, peripheral blood, peritoneal fluid, and other organs and glands. Most of these atypical manifestations have been described in Europe with *L. infantum* as the etiologic agent [16]–[18]. Although in Ethiopia coinfected patients generally present with the classical signs and symptoms [19], atypical manifestations have also been reported [20], [21]. Gastrointestinal tract and skin involvement was frequently described.

Atypical presentations of VL can easily be confused with several other opportunistic conditions that occur in HIV patients and therefore cause a diagnostic challenge. Oral and skin lesions might be misdiagnosed as Kaposi sarcoma or skin tumours. Patients with disseminated disease often have a very weak immune response and may die even before the diagnosis is established. In resource-poor settings where case detection and management according to the existing guidelines are based on clinical case definitions and a syndromic approach [7], these atypical VL presentations in HIV patients might be missed and underreported.

In *L. donovani*-transmission regions, a number of patients develop a maculopapular or nodular skin rash following successful treatment for VL. This syndrome, called post-kala-azar dermal leishmaniasis (PKDL), starts around the paranasal and perioral areas and spreads to the rest of the body. PKDL is reported in up to 50% of VL-treated patients in Sudan [22]. While most PKDL case reports from Sudan were in HIV-negative patients, a study in Ethiopia indicated that PKDL was more frequent among HIV patients, with an incidence of moderate–severe PKDL of 27.3% in HIV patients and 13.3% among non-HIV patients by the sixth month after VL treatment [23]. It presents as a different clinical presentation, showing nodular lesions that contain an abundance of parasites. In contrast to the immune-competent host where PKDL develops after treatment, in HIV patients it may occur during VL (para-kala-azar dermal leishmanias [para-KDL]). However, skin lesions concomitant with visceral disease have been reported among HIV patients with the same parasite strain isolated both from the skin and the spleen [20]. As a consequence of severe immunosuppression, distinguishing para-KDL from VL with dissemination to the skin is complicated during this kind of presentation. The possible visceralization of dermatotropic species prevalent in the region (*L. major*, *L. tropica*, and *L. aethiopica*) requires confirmation by molecular tests.

### Laboratory Diagnosis

#### Main diagnostic methods used for VL in East Africa

The commonly available serological tests in the region, the rK39 rapid diagnostic test (RDT) and the direct agglutination test (DAT), showed inconsistent and generally lower performance in comparison to the Indian subcontinent in immunocompetent VL. A meta-analysis examining the diagnostic performance of the rK39 RDT reported an average sensitivity of 79% and specificity of 85% [24]. The rK39 test's accuracy also depends on the format of the test used. The DiaMed-ITLEISH (Bio-Rad Laboratories) showed satisfactory sensitivity (85%–90%) and specificity (90%–99%) in immunocompetent patients in East Africa and performs significantly better than the Kalazar Detect (Inbios International) [25], [26]. The few studies available suggest DAT performance to be slightly better than the rK39 RDT [24], [27]. However, DAT is often not available in the public health sector in this region. MSF currently uses an algorithm with serial serological testing (rK39 followed by DAT if rK39 is negative) conducted at the first stage and invasive procedures restricted to those who remain with diagnostic uncertainty [28].

Studies on other serological tests (indirect fluorescent antibody test [IFAT], enzyme-linked immunosorbent assay [ELISA], western blotting), on urine antigen, and on molecular diagnosis are hardly available [29].

#### Diagnosis of VL in HIV-coinfected individuals

Few studies have assessed the performance of the main serological tests in HIV-positive patients in East Africa. Compared to HIV-negative individuals, sensitivity of the rK39 RDT (DiaMed-IT LEISH) was lower (77% versus 87%) among parasitologically confirmed cases in a study in Ethiopia. The sensitivity of DAT was generally higher but still lower among HIV-coinfected patients (89% versus 95%). Of interest, rK39 and DAT combined in a serial algorithm yielded a sensitivity of 98%, probably due to partly nonoverlapping sensitivity of both tests [28]. A high positive predictive value of DAT (93.8%) was found in another study, but sensitivity and specificity were not reported [30]. Other studies have also demonstrated that DAT titers in HIV-positive and HIV-negative individuals are comparable [28], [30], [31]. Overall, the performance of serological tests in HIV-coinfected individuals in East Africa is still better than what has been reported in European studies [5], [32]. While the lower sensitivities of rK39 and DAT may be due to the impact of HIV on antibody production, it has been proposed that the performance of serological tests in HIV-coinfected individuals may also depend on which infection was acquired first [5].

The suboptimal sensitivity of the commonly available serological test (rK39 RDT) explains the continued reliance on parasitological tests that can only be provided in a limited number of hospitals in the endemic areas. Some studies suggest that higher tissue parasite densities occur in HIV-coinfected patients [23], [28], [31]. Parasite detection is also often the only way to diagnose atypically localized manifestations of VL [21] and allows for further study on the strain type. Given the overall poor and unpredictable treatment response, parasitological diagnosis is also necessary to assess treatment response and to decide on the need of treatment extension or change of drugs. The standard means of parasitological diagnosis in VL entails microscopy and/or culture from spleen, bone marrow, or lymph nodes. While highly accurate, the procedure is invasive, painful, and carries the risk of potentially fatal bleeding. Due to high treatment failure and the relapsing nature of the disease, VL-HIV patients will be repeatedly exposed to these tests. Other parasite detection methods not yet thoroughly explored rely on the microscopic detection of parasites in the peripheral blood. Such methodology could help avoid invasive procedures and could be easily applied in basic laboratory settings. Studies conducted in European HIV-positive individuals infected with *L. infantum* have shown promising results when using concentrated blood (sensitivity 78% and specificity 100%) [32], [33]. A diagnostic study in which different peripheral blood concentration and visualization techniques are being evaluated is currently ongoing in Ethiopia. Preliminary data suggested high specificity but low sensitivity (close to 40%) [34]. Finally, the performance of a commercially available kala-azar latex agglutination urine antigen test (KAtex) to monitor treatment response (as test of cure) is being evaluated in Ethiopian HIV-infected patients (http://clinicaltrials.gov/show/NCT01360762).

Molecular testing (polymerase chain reaction [PCR] on peripheral blood and/or on bone marrow aspirate) has been used in first-line diagnosis for VL in HIV-coinfected individuals in European countries and Brazil and merits further exploration in East Africa [35]. Ongoing or planned diagnostic studies focusing on HIV-coinfected individuals in East Africa are summarized in Box 1.

**Box 1. VL Diagnosis and Treatment in HIV-Coinfected Individuals in East Africa: Current Knowledge and Practice and Ongoing or Planned Initiatives**

Diagnosis: Current knowledge and practice

- Lower accuracy of rK39 RDT and DAT in East Africa in the general VL patient population compared to other *L. donovani-*endemic regions (Indian subcontinent)
- The limited data on HIV-coinfected individuals suggest somewhat lower accuracy of serological tests
- Sequential diagnostic algorithms combining serological (rK39 followed by DAT) and parasitological testing achieve high accuracy with less need for invasive procedures
- Diagnosis in HIV-coinfected patients still often relies on invasive procedures for parasitological diagnosis and for monitoring of treatment response

Diagnosis: Ongoing or planned VL diagnostic studies in HIV-coinfected individuals (Ethiopia)

- Noninvasive parasitological diagnosis using peripheral blood microscopy
- Microculture inoculation of peripheral blood mononuclear cells
- Urine antigen tests: Evaluation of KAtex for diagnosis and treatment response, test of cure (TOC)
- Molecular methods: reverse transcription loop mediated isothermal amplification (RT-LAMP) assay to be evaluated in 2013 (Foundation for Innovative New Diagnostics [FIND])

### Treatment Outcomes

#### VL treatment in HIV coinfection in East Africa

Striking differences exist in VL treatment response in the general VL patient population across and within regions [36]–[38]. Higher doses of paromomycin and liposomal amphotericin B appear necessary for treatment of *L. donovani* in East Africa than in the Indian subcontinent. Of interest, within East Africa, clear differences in efficacy were seen with these drugs in between and within different countries, with the lowest cure rates noted in northern Ethiopia and Sudan. Possibly, these observations could extend to VL-HIV coinfection as well.

For decades, antimonials have been the cornerstone of VL treatment in Africa. Although these drugs still maintain good efficacy in East Africa, their use is associated with unacceptably high and potentially fatal toxicity in VL-HIV coinfection [31]. Reported death rates during antimonial treatment typically have been 4- to 10-fold higher compared to HIV-negative individuals [12], [23], [38] and have varied between 6.5% and 24.5% in a more recent study [19]. In a recent Ethiopian study, high parasitologically confirmed treatment-failure rates (30%) were observed in HIV-infected patients treated with antimonials [39]. Table 2 summarizes the studies showing the treatment outcomes of VL and VL-HIV for different antileishmanial drugs from 1998 on.

**Table 2:** Clinical studies and trials reporting treatment outcomes of HIV/VL in East Africa (1998–2013).

Reference	Study Population and Design	Treatments	Patients	Initial Cure	Mortality	Initial Failure^a^	Tolerability	Comments
Diro E, et al. [39]	Prospective study: initial treatment outcome of adult patients screened for PSP study (2011–2012)	SSG 20 mg/kg/d, 30 days	HIV+: 53	43.4%	11.3%	30.2%	5.7% SSG discontinued for safety	Requiring SSG extension: 20 (37.7%). Final outcome: 77.4% cure
Ritmeijer K, et al. [41]	Retrospective study: HIV+ adult VL patients in north Ethiopia (2010–2013), NGO program	AmBisome30 mg/kg total+MF 100 mg po/d −28 days	Total: 111	81.1%	9.0%	6.3%^b^	NA	
			VL relapse: 54	83.3%	3.7%	11.1%^b^		
Hailu W, et al. [44]	Retrospective study. All patients treated with antimonials (2008–2009) at teaching public hospitals	Glucantime 20 mg/kg/d for 30 days (N- Ethiopia)	Total: 30	73.3%	10%	16.7%	2 pancreatitis and 1 renal failure	
			- HIV+: 12	58.3%	NA	NA	NA	2/3 (66.7%) relapse by 6 months among the HIV coinfected (only 25% seen by month 6
			- HIV−: 14	92.9%	NA	NA	NA	
		Glucantime 20 mg/kg/d for 30 days (S-Ethiopia)	Total: 24	100%	0	0	NA	No relapse in HIV−
Ritmeijer K, et al. [38]	Retrospective study. Severely sick or HIV+ adult VL patients (2007–2008), NGO program in N- Ethiopia	AmBisome 30 mg/kg^c^	HIV+: 195	59.5%	6.7%	32.3%^b^	NA	21.5% on ART at VL diagnosis; 29/43 (67%) with CD4 less than 200
			- PVL: 116	74.1%	7.8%	16.4%		
			- Relapse: 79	38.0%	5.1%	55.7%		
			HIV−: 94	92.6%	6.4%	None^b^	NA	
			- PVL: 84	91.7%	7.1%	0		
			- Relapse: 10	100%	0	0		
		SSG (30–40 d) 20 mg/kg/d as rescue therapy	HIV+ failing AmBisome^d^: 58	70.7%	15.5%	1.7%	5/63 (7.9%) unable to tolerate SSG	
Hurissa Z, et al. [19]	Retrospective record analysis. All admitted adult VL patients (2006–2008). Two public hospitals in N- Ethiopia	SSG: 20 mg/kg/d for 30 days AmBisome: 3 mg/kg 6–10 days (only critically ill)	Adults: 241	84.6%	10%	5.4%	NA	Case fatality in HIV coinfected high SSG (24.5%) AmBisome (7.7%)
			- HIV+: 92	68.5%	17.4%	14.1%		
			- HIV−: 149	94.6%	5.4%	0		
Ritmeijer K, et al. [31]	Randomized controlled trial, nonblinded in N-Ethiopia. Male migrant workers. PVL (546); relapse (34)	MF 100 mg/d for 28 days	Total: 290	88.3%	2.1%	7.9%	Severe vomiting: 14/290 (4.8%)	At sixth month: Relapse: 10.3%
			- HIV+: 63	77.8%	1.6%	17.5%		- HIV+: 25.4%
			- HIV−: 131	93.8%	0.8%	4.5%		- HIV−: 4.6%
			- Unknown: 96	87.5%	4.2%	6.3%		
		SSG 20 mg/kg IM for 30 days	Total: 290	87.6%	9.7%	0.7%	Severe vomiting: 27/290 (9.7%); mainly HIV+	Relapse: 2.4%
			- HIV+: 44	90.1%	6.8%	2.3%		- HIV+: 11.4%
			- HIV−: 137	94.9%	2.9%	0.7%		- HIV−: 0.0%
			-Unknown: 109	77.1%	19.3%	0.0%		
Lyons S, et al. [65]	Retrospective study, NGO program in N-Ethiopia (1998–2000)	No info (SSG only available drug)	Total: 791	81.5%	18.5%	NA	NA	
			- HIV+: 49	73.5%	26.5%			
			- HIV− : 164	93.3%	6.7%			
Ritmeijer K, et al. [23]	Randomized controlled trial, nonblinded, 1998–1999. Only PVL adults included. NGO program; N-Ethiopia	SSG versus Pentostam	All primary VL. Total: 199	75.4%	24.1%	NA	Vomiting Total:	Relapse: 3/114 (2.6%)
			- HIV+: 27	63%	33.3%		44.4%	2/12 (16.7%)
			- HIV−: 112	96.4%	3.6%		35.7%	1/83 (1.2%)

Abbreviations: PVL, primary visceral leishmaniasis; HIV−, HIV negative; HIV+, HIV positive; NA, not available; N-Ethiopia, north Ethiopia; S-Ethiopia, south Ethiopia; NGO, nongovernmental organization; PSP, pentamidine secondary prophylaxis; SSG, sodium stibogluconate, IM, intramuscular.

a clinically defined unless otherwise stated;

b parasitologically confirmed;

c median dose used;

d subgroup of the 195 HIV+ patients treated with liposomal amphotericin B (mentioned above).

In the search for a safer alternative, liposomal amphotericin B has increasingly been explored in East Africa. While studies have consistently reported an excellent tolerability, cure rates in HIV-infected individuals have been rather disappointing in this continent. At a total dose of 30 mg/kg, around 16% of primary VL and 56% of VL relapse cases demonstrate parasitological failure in northern Ethiopia [38]. This is in clear contrast with a report from India in which high (100%) cure rates were achieved at a total dose of 20 mg/kg [40]. Current WHO guidelines recommend a cumulative dose of 40 mg/kg, with 8 to 10 doses of 3–5 mg/kg taken daily or intermittently, for VL-HIV coinfection in East Africa [7], although this has not yet been evaluated in the region.

Miltefosine has been evaluated in only one clinical trial in Ethiopia [31]. In comparison with antimonials, it was found safer but less effective, with 17.5% parasitological treatment failure. Interestingly, a compassionate use of miltefosine in combination with liposomal amphotericin B (at 30 mg/kg total dose) in 111 HIV-coinfected VL patients seems to suggest substantially higher cure rates and lower failure rates both in primary VL and VL relapse [41]. Based on this emerging evidence, a clinical trial is planned to start in northwest Ethiopia by the end of 2013, evaluating in parallel two treatment options: (1) combination therapy: miltefosine (2.5 mg/kg per day) for 28 days combined with liposomal amphotericin B (6 doses of 5 mg/kg; total dose 30 mg/kg) and (2) a high dose of liposomal amphotericin B (8 doses of 5 mg/kg; total dose 40 mg/kg).

Whereas the combination of antimonials and paromomycin (for 17 days) is now recommended by WHO as first-line treatment in immunocompetent individuals in East Africa, experience with it as a first-line treatment for HIV-coinfected individuals is limited [42]. This regimen is now used in some programs for VL-HIV coinfected patients as a second-line treatment (in case of intolerance or failure of liposomal amphotericin B) [43] or as a first-line treatment if access to and availability of liposomal amphotericin B is limited. Current national and international treatment recommendations for VL-HIV coinfection are summarized in Table 3.

**Table 3:** Treatment recommendations for VL and HIV in different guidelines used in the East Africa region.

Guideline	First-line Treatment	Second-line Treatment	Indications for ART
WHO (2010)	Amphotericin B lipid formulations, total dose of 40 mg/kg; given as 3–5 mg/kg daily or intermittently for 10 doses (days 1–5, 10, 17, 24, 31, and 38)	Pentavalent antimonials (in areas without drug resistance)	All VL-HIV patients
MSF in Sudan, South Sudan, and Ethiopia (2012)	Liposomal amphotericin B, 30 mg/kg (given as 5 mg/kg on alternate days for 6 doses)+Miltefosine 100 mg (divided in two doses) for 28 days	SSG 20 mg/kg/day for up to 30 days plus paromomycin 15 mg/kg/day for 17 days	All VL-HIV patients
National guidelines			
Ethiopia (2013)	Liposomal amphotericin B, 40 mg/kg total dose; given as 5 mg/kg on day 1–5, 10, 17, and 24	Pentavalent antimonials	All VL-HIV patients
Sudan (2013)	Liposomal amphotericin B, 3 mg/kg/day for 10 to 14 days	Not specified	Not specified
South Sudan (2012)	Liposomal amphotericin B, 40 mg/kg total dose; given as 3–5 mg/kg on days 1–5, 10, 17, 24, 31, and 38	Pentavalent antimonials	Not specified
Kenya (2012)	Liposomal amphotericin B (higher dose may be required, routinely recommended total dose is 30 mg/kg)	Amphotericin B	All VL-HIV patients
Uganda (2007)	Liposomal amphotericin B, 3 mg/kg/d for 7 days	Amphotericin B, 1 mg/kg every other day for 30 days. Miltefosine, 100 mg (2.5 mg/kg)/d for 28 days	All VL-HIV patients

Abbreviations: WHO, World Health Organization; MSF, Médecins Sans Frontières.

#### Risk of relapse and secondary prophylaxis in VL-HIV coinfection

Given the high rates of lost to follow-up in most reported studies (often above 50%) [19], [23], [44], reliable data on the risk of relapse in VL-HIV-coinfected individuals are very scarce. In the most complete study, the reported risk of relapse at six months varied between 25.4% for the miltefosine group and 11.4% for the sodium stibogluconate (SSG) group [31]. Another study estimated a one-year relapse risk of close to 20% for individuals with primary VL and CD4 cell counts of around 200 cells/µL and around 60% for those with multiple previous VL episodes and CD4 cell counts below 100 cells/µL [45]. However, the potential bias caused by the high proportion of patients not receiving ART who were lost to follow-up in this study compromises the generalizability of these estimates. In line with European data, use of ART was associated with an estimated 50% reduced risk of relapse in this study. VL relapse was also associated with persistently low CD4 counts while on ART.

A recent systematic review, mainly containing data on *L. infantum* in Europe, suggested that secondary prophylaxis could reduce the risk of relapse in VL-HIV coinfection by at least 50% [46], [47]. Whereas secondary prophylaxis against VL is indeed recommended by WHO for VL-HIV coinfection in areas with zoonotic transmission, this is less clear when transmission is human to human (antroponotic transmission) [5]. In such a situation, use of any of the few available VL treatment drugs for secondary prophylaxis carries the risk of emergence and spread of drug resistance. Pentamidine has been proposed for secondary prophylaxis in such a situation, since it exerts antileishmanial effect, is currently not used for VL treatment, and has been found to be relatively safe in a prophylactic dose [48]–[50]. An open-label multicentre clinical trial recruiting HIV coinfected adults at high risk of VL relapse is currently ongoing in Ethiopia to evaluate the feasibility, safety, and effectiveness of monthly intravenous administration of pentamidine (4 mg/kg) for a period of 12 to 18 months. Main outcome data are expected before the end of 2014 (http://clinicaltrials.gov, NCT01360762).

#### Antiretroviral treatment in VL-HIV coinfection

Within Europe, the widespread use of ART in VL-endemic areas has had a major epidemiological effect on VL-HIV coinfection, with a pronounced reduction of new cases and prolonged disease-free survival for established VL-HIV coinfection. The effect on relapse has been more modest, with an estimated 50% reduction. Although ART has now been scaled up in most African countries, access to ART in the generally remote and underresourced VL-endemic areas remains challenging. Only in Ethiopia do all (major) VL treatment sites provide ART. Usually, ART is initiated as soon as the patient is clinically stable, typically during the second week after initiation of VL treatment.

There is only one retrospective study on the effect of ART on survival in VL-HIV coinfection in East Africa, which did not demonstrate an effect [51]. However, this might be due to the high proportion of lost to follow-up patients who were interrupting their ART. It was also not assessed whether HIV viral suppression was achieved [52].

Overall and in clear contrast with TB-HIV, the immune reconstitution inflammatory syndrome (IRIS) appears to be an exception in VL-HIV coinfection. Only a handful of clear-cut case reports of (ART-associated) VL-IRIS have been reported at the global level [53]–[58]. Whether the overall dampening effect of the *Leishmania* parasite on the immune system could relate to this requires further study. The lack of a clear case definition of IRIS in VL-HIV could further add to this lack of reporting. In East Africa, a few cases of VL have been reported after ART initiation (including seroconversion), but it is not clear to what extent these are true “unmasking” IRIS cases [45]. While PKDL is assumed to be an immune-mediated condition [59], in HIV patients on ART, it may indicate IRIS [58], [60]. Two cases were reported from Ethiopia with exacerbation of skin lesions after ART initiation, described as PKDL or PKDL-like lesions [20]. Detailed prospective studies are required to characterize IRIS in VL-HIV coinfection, with a special focus on skin manifestations.

With the global scaling-up of ART, HIV-1 protease inhibitors (PIs) are increasingly available in VL-HIV-endemic regions, including regions in Africa. Several lines of evidence suggest that HIV-1 protease inhibitors might directly exert antiparasitic effects, including effects against *Leishmania*. One potential approach could consist of using PI-based ART instead of non-nucleoside reverse transcription inhibitor (NNRTI)-based ART in VL-HIV-endemic regions. However, with detailed animal or clinical studies lacking, additional research is required before HIV-1 PIs should be taken forward towards this goal [61].

### The Role of VL-HIV Coinfections as Reservoirs for Transmission of *L. donovani*

The infectiousness of HIV-coinfected patients to sand flies in endemic areas of anthroponotic foci has not been studied. However, coinfected patients were found to have higher tissue parasite loads and higher rates of PKDL [23], [28] potentially acting as reservoirs. Given the high rate of treatment failure and risk of relapse [19], [45] and the associated repeated and prolonged exposure to antileishmanial drugs, HIV-coinfected individuals are also at increased risk of developing drug resistance and could possibly serve as a source of resistant parasites. Ideally, xenodiagnosis studies should be conducted in East Africa as well in order to better define the epidemiological impact of HIV coinfection in this region.

## Discussion and Conclusions

As a neglected disease, the significance of *Leishmania* infection in HIV patients was recognized late. The poorest segments of the population, such as migrant daily laborers, are affected. Thus, the diagnostic and treatment challenges of VL-HIV coinfection have continued to date, especially in East Africa [5], [11].

The control of VL requires a combined effort at vector control, improved living standards and case detection, and early treatment [13]. The feasibility of transmission prevention methods like impregnated bed nets, blankets, and protective clothing should be evaluated. In the case of VL-HIV, targeting the most at-risk population groups (e.g., the migrant population) and improving their awareness through health education and counseling for both diseases should be pursued. Health professions should also be regularly updated about VL.

Case detection and management is especially challenging for HIV-infected persons. To have a larger impact on VL-HIV coinfection, a comprehensive and multipronged approach will be required. Better diagnostic and curative options would help to improve case detection and patient management. As with other HIV-associated coinfections (for instance, tuberculosis and cryptococcal meningitis), preventive strategies should be explored. As the experience in Europe testifies, the preventive effect of large-scale introduction of ART merits exploration. Although the current WHO guidelines now recommend early ART initiation [62], the most challenging part will be early HIV diagnosis and retention in care in a typically highly mobile and difficult to reach population.

Diagnosing VL in HIV patients relies mainly on parasite detection from tissue aspiration. This is due to the insufficiently sensitive serological tests that also do not help to diagnose relapsed cases, which are very common in HIV patients. However, tissue aspiration is associated with potential risk of fatal bleeding that requires experience and health facilities equipped to handle this potential complication. Thus, a simple-to-use but reliable antigen-detection diagnostic procedure or tool that can be applicable for treatment monitoring and diagnosing relapses is urgently required.

For VL-HIV case management, field experience favors the use of the following combination treatment: liposomal amphotericin B and miltefosine [41] followed by secondary prophylaxis [7], [47], [63] and ART. However, studies are urgently needed to strengthen the evidence for this treatment and improve its outcome in patients in field conditions in East Africa. To date, there is no clear evidence regarding the interaction of ARV drugs and antileishmanial medications. Basic research to understand the immunological interaction of the two infections, as well as the immune modulatory effects of drugs, may ultimately help to improve the management of the coinfection.

Tackling VL-HIV coinfection in the long run, along with combating VL in general, will strongly depend on the strength and commitment of the national programs. However, to date, the national programs still rely on substantial external support in most East African countries. Moreover, a strong link with the national HIV program will be needed for efficient and integrated program management. Though the HIV prevalence is declining in Ethiopia, the unstable socioeconomic and political situations in South Sudan and the high population migrations in the region warrant continuous effort for the control of both diseases.

**Box 2. Key Learning Points**

- Northwest Ethiopia has the highest known burden of VL-HIV coinfection rates in the world.
- VL-HIV coinfection is associated with diagnostic and treatment challenges that still need additional research.
- Atypical clinical presentations and poor performance of rapid serological tests among the HIV co-infected VL patients poses a diagnostic challenge.
- There is a high rate of treatment failure and relapse of VL among HIV-coinfected patients.
- Combination treatment with liposomal amphotericin B and miltefosine followed by secondary prophylaxis and ART seems to be a promising standard of care that needs clinical trials.

**Box 3. Five Key Papers in the Field**

- Hurissa Z, Gebre-Silassie S, Hailu W, Tefera T, Lalloo DG, et al. (2010) Clinical characteristics and treatment outcome of patients with visceral leishmaniasis and HIV co-infection in northwest Ethiopia. Trop Med Int Health 15: 848–855. doi: 10.1111/j.1365-3156.2010.02550.x
- ter Horst R, Tefera T, Assefa G, Ebrahim AZ, Davidson RN, et al. (2009) Field evaluation of rK39 test and direct agglutination test for diagnosis of visceral leishmaniasis in a population with high prevalence of human immunodeficiency virus in Ethiopia. Am J Trop Med Hyg 80: 929–934.
- ter Horst R, Collin SM, Ritmeijer K, Bogale A, Davidson RN (2008) Concordant HIV infection and visceral leishmaniasis in Ethiopia: the influence of antiretroviral treatment and other factors on outcome. Clin Infect Dis 46: 1702–1709. doi: 10.1086/587899
- Ritmeijer K, ter Horst R, Chane S, Aderie EM, Piening T, et al. (2011) Limited effectiveness of high-dose liposomal amphotericin B (AmBisome) for treatment of visceral leishmaniasis in an Ethiopian population with high HIV prevalence. Clin Infect Dis 53: e152–e158. doi: 10.1093/cid/cir674
- Ritmeijer K, Dejenie A, Assefa Y, Hundie TB, Mesure J, et al. (2006) A comparison of miltefosine and sodium stibogluconate for treatment of visceral leishmaniasis in an Ethiopian population with high prevalence of HIV infection. Clin Infect Dis 43: 357–364. doi: 10.1086/505217
